# Efficacy Study of Anti-Endomysium Antibodies for Celiac Disease Diagnosis: A Retrospective Study in a Spanish Pediatric Population

**DOI:** 10.3390/jcm8122179

**Published:** 2019-12-11

**Authors:** María Roca, Ester Donat, Natalia Marco-Maestud, Etna Masip, David Hervás-Marín, David Ramos, Begoña Polo, Carmen Ribes-Koninckx

**Affiliations:** 1 Celiac Disease and Digestive Immunopathology Unit, Instituto de Investigación Sanitaria La Fe, 46026 Valencia, Spain; donat_est@gva.es (E.D.); natamarco93@gmail.com (N.M.-M.); Masip_etn@gva.es (E.M.); polo_beg@gva.es (B.P.); ribes_car@gva.es (C.R.-K.); 2 Pediatric Gastrohepathology Unit, Hospital Universitario y Politécnico La Fe, 46026 Valencia, Spain; 3 Statistics Unit, Instituto de Investigación Sanitaria La Fe, 46026 Valencia, Spain; bioestadistica@iislafe.es; 4 Pathology Service, Hospital Universitario y Politécnico La Fe, 46026 Valencia, Spain; david.ramos@uv.es

**Keywords:** Celiac disease, anti-endomysium antibodies, anti-tissue transglutaminase antibodies, pediatric population

## Abstract

The aim of this study was to assess the efficacy of anti-endomysium antibodies (EMA) as a serological marker for celiac disease (CD) diagnosis in a pediatric population. A retrospective study of pediatric patients who underwent a CD serological markers study: EMA and anti-tissue transglutaminase antibodies (anti-TG2). Clinical symptomatology, degree of histological lesion, human leukocyte antigen (HLA) haplotype compatible with CD (HLA DQ2 and/or DQ8), and final diagnosis were taken into account. We included 445 patients who were classified in two groups according to the final diagnosis. Group 1: 232 children with CD, 91.4% of whom exhibited small intestinal villous atrophy, 228 being EMA-positive and four EMA-negative. Group 2: 213 children with a non-CD diagnosis, 212 EMA negative and one EMA positive. Both antibodies, EMA and anti-TG2, reached similar sensitivities, 98% and 99% respectively, while EMA had a higher specificity (99%) than anti-TG2 (93%). By using both markers combined, compared to using anti-TG2 alone, 5.7% of patients are better diagnosed. However, when we compare the efficacy of EMA and anti-TG2 in asymptomatic and symptomatic patients, the sensitivity of EMA is 98% irrespective of symptoms, thus higher than for anti-TG2 ≥10 × upper limit of normal (ULN) (respectively 77% and 84%). Our results support the use of EMA to increase CD diagnostic accuracy in a non-biopsy approach, especially in asymptomatic children.

## 1. Introduction

Celiac disease (CD) is an immune-mediated systemic disorder triggered by the consumption of gluten and related prolamins in genetically susceptible individuals. CD is associated with the human leukocyte antigen (HLA) DQ2 and DQ8 haplotypes. It is characterized by a variable combination of gluten-dependent clinical manifestations, CD-specific antibodies, and enteropathy [1].

*The European Society for Paediatric Gastroenterology, Hepatology and Nutrition* (ESPGHAN) guidelines published in 2012, allow for a diagnosis of CD without biopsies in children and adolescents with symptoms and levels of immunoglobulin A against anti-tissue transglutaminase antibodies (anti-TG2) >10 times the upper limit of normal (ULN), confirmed by anti-endomysium antibodies (EMA) and positivity for HLA DQ2 and/or DQ8 [1]. In these cases, the enteropathy, detected by a small intestinal biopsy (SIB), is an additional diagnostic element but is not an essential criterion. Thus, CD antibodies are considered highly specific, especially in children. Moreover, EMA testing reaches a higher specificity (98%–100%) when it is carried out by experienced technicians, therefore EMA is considered the reference standard for CD-specific antibodies. A recent multinational prospective study (ProCeDE) [2] validates this non biopsy approach in children presenting clinical symptoms whenever anti-TG2 levels are >10 × ULN and with positive EMA in a second blood sample, thus supporting the use of EMA as a confirmatory test when CD diagnosis is performed without biopsy. The authors also conclude HLA does not improve the diagnostic accuracy if the abovementioned criteria are met. Similarly, Wolf and colleagues [3] observed in a prospective study that testing for EMA and HLA did not increase the positive predictive value (PPV) in cases with anti-TG2 >10 × ULN. However, the majority of patients were included based on prior positive anti-TG2 tests, and because of the patient’s preselection the specificity of EMA is lower (94%) than commonly described. Based mainly on these two studies, the 2019 ESPGHAN guidelines state that the non-biopsy approach is safe in children with anti-TG2 >10 × ULN and positive EMA without the need for HLA assessment [4]. 

An evidence-based review of the accuracy of serological markers for CD diagnosis reports an overall slightly better sensitivity for anti-TG2 compared to EMA, and conversely a higher specificity for EMA (98%) compared to anti-TG2 (≥90%–95%) [5]. However, the specific role of EMA in combination with anti-TG2 has been addressed by a limited number of studies and is still a matter of debate. 

The aim of our study is to assess the contribution of EMA to the accuracy of serology-based CD diagnosis in the non-biopsy approach, not only in symptomatic, but also in asymptomatic patients.

## 2. Patients and Methods

### 2.1. Study Design and Participants

We have retrospectively evaluated pediatric patients, aged 0.8 to 15 years, who were referred to the Pediatric Gastroenterology and Hepatology Unit of La Fe University Hospital between 2009 and 2017, for serological evaluation because of clinical symptoms suggesting CD or as screening in at risk groups. Only those in whom serological CD markers and total serum IgA levels were available were considered for statistical analysis.

Additional inclusion criteria were: Determination of EMA and anti-TG2 antibodies from the same serum sample, serum samples must be collected no earlier than 3 weeks before the biopsy, if performed, and patients were on a gluten-containing diet at the time of biopsy and blood sampling. Patients who did not have a final diagnosis and/or their histopathological study was not valid for interpretation and/or had an IgA deficiency, were excluded from the study. CD diagnosis was based on ESPGHAN 1990 and 2012 criteria [1,6] 

Data on clinical symptoms, final diagnosis, degree of histological lesion, and HLA genotyping (DQ2 and/or DQ8) were obtained from the clinical files.

The present study was approved by the Ethics Committee of La Fe University Hospital. The number of ethical approval: 2017/0002.

### 2.2. Methodology

#### 2.2.1. Serology

EMA antibodies were routinely tested by an indirect immunofluorescence method (IFI) using monkey esophagus sections (Biosystems^®^, Barcelona, Spain). The test serum samples were diluted 1:5 and incubated for 30 minutes with anti-human immunoglobulin A conjugated with fluorescein isothiocyanate (FITC) (Dako^®^, Glostrup, Denmark) 1:20 dilution. Vectashield mounting medium for fluorescence (Vector Laboratories, Burlingame, CA, USA) was added before carefully covering with a coverslip. Slides were blindly examined using a Motic Trinocular BA400 with filter for FITC microscope (20×) by two experienced technicians with more than ten years of experience reading EMA results. A positive result was recorded if the connective tissue surrounding the muscle cells was brightly fluorescent, forming a honeycomb pattern. 

IgA anti-TG2 detection was performed in an automated way by fluoroenzymeimmunoassay according to the manufacturer’s instructions (EliA CeliKey Phadia-thermofisher^®^, Uppsala, Sweden). Values higher than 7 U/mL were considered positive for IgA anti-TG2 antibodies as established by the manufacturer. 

#### 2.2.2. Histopathological Assessment of Intestinal Mucosa

SIB samples were formalin-fixed and paraffin-embedded, stained with hematoxylin-eosin, and studied under an optical microscope. Samples were interpreted and classified according to the Marsh-Oberhuber classification [7].

#### 2.2.3. HLA

HLA typing was performed using a polymerase chain reaction (PCR) technique for DRB1/DQA1/DQB1 alleles’ detection. HLA class II (loci DRB1/3/4/5, DQA1, DQB1) low- or high-resolution genotyping was performed by polymerase chain reaction with sequence-specific primers [8,9]. Genomic DNA was isolated from nucleated cells by digestion with proteinase K [10].

#### 2.2.4. Statistical Analysis

Data were summarized by their means and standard deviation (SD) in the case of continuous variables, and absolute and relative frequencies in the case of categorical variables. Sensitivity and specificity, PPV and negative predictive value (NPV) were also estimated; positive likelihood ratio (LR+) and negative likelihood ratio (LR−) and diagnostic odds ratio (DOR) were computed for both antibodies. To assess the possible contribution of the predictive improvement with the combined use of EMA + anti-TG2 regarding the use of anti-TG2, Integrated Discrimination Improvement (IDI) and Net Reclassification Improvement (NRI) indexes were estimated [11]. The statistical significance level was set at a *p*-value less than 0.05. All of the statistical analyses were performed using R software (version 3.4.0). (R Foundation for Statistical Computing: Vienna, Austria).

## 3. Results

**Figure 1:**
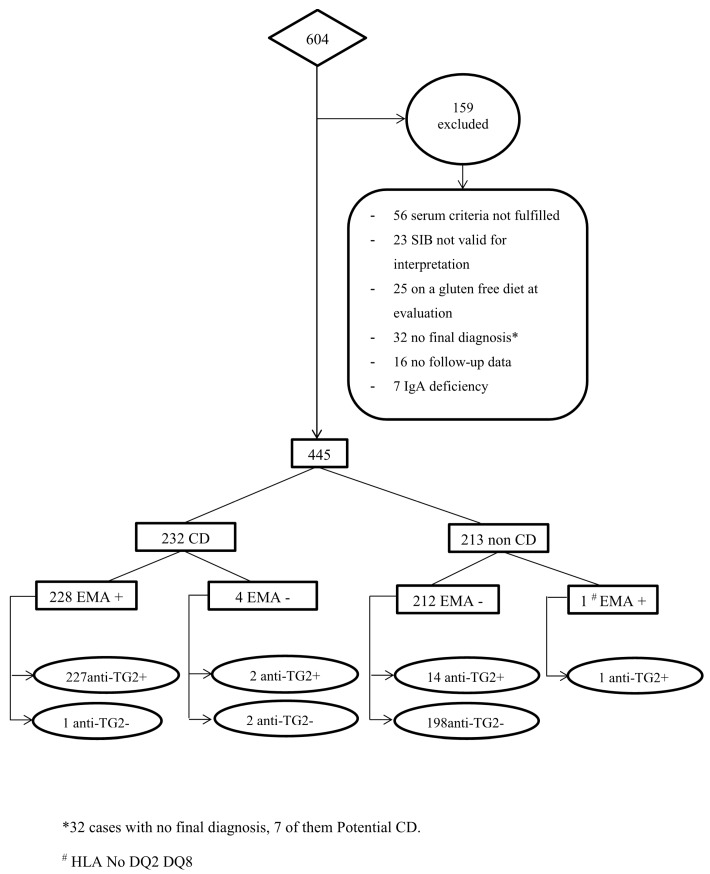
Flow chart of patients included in the study. SIB: Small intestinal biopsy; IgA: Immunoglobulin A; CD: Celiac disease; EMA: Anti-endomysium antibodies; Anti-TG2: Anti-tissue transglutaminase antibodies.

Out of 604 patients referred to the Pediatric Gastroenterology and Hepatology Unit for CD serological evaluation because of suggestive clinical symptoms or as screening in at risk groups, 445 (250 girls and 195 boys) were included in the study. A further 159 children were excluded because either a final diagnosis was missing, anti-TG2 and EMA results from the same serum sample were not available, a gluten free diet (GFD) had been started, the SIB was not optimal for histological evaluation (whenever the SIB was mandatory), and/or patients were not followed in our Unit (Figure 1). Patients with IgA deficiency were also excluded. Seven of them could be considered as potential CD patients, according to ESPGHAN criteria, namely due to the presence of anti-TG2 and EMA antibodies, compatible HLA and only minor histological lesion (M0 or M1) [1], but as their final diagnoses were uncertain, they were not considered for the statistical analysis.

According to the final diagnosis, 445 patients were classified in two groups: Children with a firm diagnosis of CD (Group 1) and children in whom the CD diagnosis had been ruled out (Group 2) (Supplemental Table S1).

Overall, SIB was performed in 253 children; in 242 (95.7%) by Watson-Crosby capsule, and in 11 by endoscopy. We present data from a historical cohort in which the majority of children were biopsied using the Crosby capsule; even so, those patients with uncertain diagnosis were re-biopsied by upper digestive endoscopy. 

**Figure 2:**
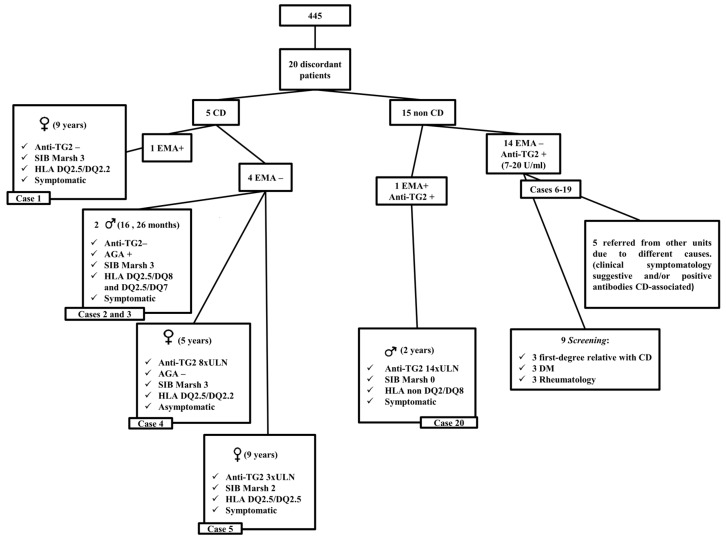
Twenty of the 445 cases with discordant serology results. CD: celiac disease; SIB: Small intestinal biopsy; EMA: Anti-endomysium antibodies; Anti-TG2: Anti-tissue transglutaminase antibodies; HLA: Human leukocyte antigen; ULN: Upper limit of normal; DM: Diabetes mellitus.

Group 1 comprised 232 children (age 5.6 ± 3.9 years, mean ± SD) with a final CD diagnosis, 212 (91.4%) had villous atrophy Marsh 3 in histological assessment, and 19 had Marsh 2 (8.2%); 228 were EMA-positive and four were EMA-negative. Out of 228 children positive for EMA, 227 had anti-TG2 levels >7 U/mL and only one patient (case 1; Figure 2) had anti-TG2 levels below ULN (0.2 U/mL). Regarding clinical symptoms, 49 were asymptomatic, whereas 179 had one or more of the following symptoms: Diarrhea, abdominal pain, abdominal distension, vomiting, constipation, failure thrive, growth failure, short stature, asthenia, anorexia, iron deficiency, anemia, intestinal malabsorption syndrome, hypertransaminemia, headache, urticaria, dermatitis herpetiformis, mouth aphthae, irritability, and fatigue or lack of energy.

Out of the four children with CD diagnosis and negative-EMA, two cases (2 and 3), aged 16 and 26 months, respectively, had anti-TG2 levels below the ULN, were DQ2-positive (DQ2.5/DQ8 and DQ2.5/DQ7), had diarrhea and growth failure, and the histological lesion was Marsh 3. Both had positive antigliadin antibodies (AGA) or deamidated gliadin peptide antibodies (anti-DGP) and symptoms improved after the GFD. In addition, case 3 had anti-TG2 above cut of level in a previous sample. Two other patients (cases 4 and 5), five and nine years old, respectively, had anti-TG2 8 × ULN and 3 × ULN, respectively, and carried the HLA DQ2 genotype (DQ2.5/DQ2.2 and DQ2.5/DQ2.5); the first one had in the histological findings a Marsh 2 lesion and was asymptomatic; while case 5 had Marsh 3, referred to abdominal pain, and had a positive EMA result three months before the biopsy. 

Group 2 comprised 213 non-CD patients (age 6.7 ± 4.1 years, mean ± SD), one was EMA-positive in one single sample. Out of 212 EMA negative children, 198 had anti-TG2 < ULN, while 14 children (cases 6–19) showed anti-TG2 levels 1–2 × ULN (7–20 U/mL). Out of 14 children, seven were asymptomatic and seven showed one or more of abovementioned symptoms (Supplemental Table S1). Out of these 14 transient anti-TG2-positive children, 10 were HLA-compatible (no HLA data available from the remaining four patients). Since they had repeated negative-EMA and very low anti-TG2, which all normalized on follow-up while on a gluten-containing diet (several measurements), we consider these patients to be false positive for anti-TG2. 

The non-CD child (case 20) with positive EMA showed anti-TG2 levels 10 × higher than the ULN (>128 U/mL), but no histological lesion of the intestinal mucosa (Marsh 0). He had a *Rotavirus* infection a few days prior to the serology test, was HLA DQ2/ DQ8-negative, and had a clinical history of long-term diarrhea that resolved after cow´s milk protein was excluded from the diet. He was followed up at our outpatients clinic for nine years, with permanent seroconversion to negative antibodies, normal nutritional status (90 percentile for weight and height), and absence of symptoms (Figure 2). 

Finally, on the whole, 195 patients in our study had anti-TG2 >10 × ULN, and in all of them EMA was positive. 

It is worth noting that the seven potential CD children with positive EMA showed anti-TG2 levels >7 U/mL (between 2 × ULN and >10 × ULN), they had Marsh 0–1, and all carried the HLA DQ2 genotype. Of them, three children were CD first-degree family member; another two had type 1 diabetes mellitus, one had scleroderma, and in the last one serology coincided with an episode of bronchiolitis. On follow-up, all seven cases referred no CD related symptoms and had permanent negative serological CD markers while on a gluten-containing diet. 

### 3.1. Sensitivity and Specificity 

EMA sensitivity and specificity reached 98% and 99%, respectively. For anti-TG2 (cut-off value of 7 U/mL) sensitivity was 99% and specificity was 93%. We show LR+, LR−, PPV, NPV, and DOR in Table 1; PPV and LR+ for EMA being higher (99% and 209.3) than for anti-TG2 (94% and 14). Moreover, we found a high DOR, i.e., 12,084, showing a strong association between test positivity for EMA and CD diagnosis.

### 3.2. IDI and NRI

The estimation of the IDI and NRI values showed that the combined use of both markers does provide a statistically significant improvement in predictive capacity. For IDI (*p* = 0.002), the estimate was 0.057, with a 95% confidence interval between 0.022 and 0.093. The estimate for NRI (*p* = 0.002) was 0.057, with a 95% confidence interval between 0.022 and 0.092. In both cases, the value is positive, which indicates an improvement in the predictive capacity, i.e., 5.7% of patients are more accurately diagnosed by using both markers as compared to using anti-TG2 alone.

### 3.3. EMA Efficacy for CD Diagnosis 

The diagnostic accuracies for different criteria combinations are shown in Table 2. 

If ESPGHAN criteria for a non-biopsy approach had been applied in those cases who had all the data available (*n* = 284), 132 CD patients would have been correctly detected, with no false positive diagnosis; thus, the PPV value would have been 100%. Moreover, we found 71 CD patients who did not fulfill these criteria.

If HLA is excluded, sensitivity and specificity of the combined approaches remain similar to the above-reported results. However, if HLA and symptoms are excluded, sensitivity increases (82%). 

Following the 2012 criteria, in our sample the non-evaluation of the EMA hardly varies the specificity, but it contributes to a greater sensitivity. However, when the diagnosis of CD is made in asymptomatic patients, without knowing their HLA, the EMA increases the diagnostic sensitivity from 77% to 98%.

## 4. Discussion

We performed a retrospective study evaluating the performance of EMA and anti-TG2 for CD diagnosis in a pediatric population. Although both showed a high sensitivity and specificity as CD diagnostic tools, EMA had a higher specificity (99%) and a slightly lower sensitivity (98%) as compared to anti-TG2 (93%, 99%). Our results are in line with a wide evidence-based review, carried out by the ESPGHAN CD diagnosis working group in 2012 [5]. When comparing the role of EMA and anti-TG2 separately in asymptomatic and symptomatic patients, the sensitivity of the EMA is higher in both groups.

In our results, out of 232 patients with CD diagnosis and positive EMA, just one case (case 1) was anti-TG2 negative (0.20 U/mL). The diagnosis was based on the presence of symptoms (short stature, abdominal pain, and constipation), positive EMA and, although at the moment of the SIB anti-TG2 was negative, the child have had a positive anti-TG2 (3 × ULN) four months before the biopsy, and a Marsh 3 histological lesion. Additionally, in this patient, HLA was DQ2 and clinical improvement after gluten withdrawal was confirmed. Therefore, given the family was aware that CD was suspected, we might speculate a GFD was started prior to the SIB. Similar results have been found in another publication [12], reporting one anti-TG2-negative and EMA-positive CD patient out of 169. Thus, in both studies, only one patient [12] or three patients (in our study) with CD would have remained undetected if only anti-TG2 had been used for diagnostic approach as a screening test.

In the CD patients group, 4/232 were negative for EMA. In these four cases, even when applying the 2012 ESPGHAN criteria, performing the SIB would have been mandatory (negative anti-TG2 or <10 × ULN). Out of these four children, in two of them (cases 2 and 3) we could eventually attribute the negativity of EMA and anti-TG2 to their young age, 16 and 26 months, respectively [13]. Indeed, AGA emerge on average 11 months earlier than anti-TG2 and EMA, which both appear following a common pattern, not surprisingly because they are the same antibodies, but detected using different methods. AGA measurement was evaluated in some historical patients, anti-DGP not being available at that time. However, the use of AGA is nowadays not recommended as serological marker for CD diagnosis because of its low specificity; moreover, they have been surpassed by anti-DGP, although these perform worse overall than anti-TG2 and EMA, and thus their use is currently not recommended for initial testing [1]. 

In a large proportion of children with positive antibodies, these are only transiently positive, disappearing spontaneously without changes in gluten exposure [14,15,16]. Furthermore, it is not uncommon that the CD-associated antibodies emerge, disappear, and re-emerge after a short while. Thus, EMA negativity in these CD patients could be explained by the natural fluctuation of antibodies in serum [17]. However, in a recent prospective study, follow-up serology of infants at risk showed an anti-TG2-positive result in all cases who developed CD before three years of age, although the total number of CD patients was only 80 [18]. Case 5 had positive EMA and anti-TG2 three months previous to the SIB, and the parents were informed of the suspicion for CD. At the time of the SIB, EMA was negative and anti-TG2 levels had decreased. These facts, together with a SIB with a Marsh 2 lesion, support the conclusion that the girl was most likely on a gluten free diet prior to the SIB and diagnosis. 

From the non-CD patients group, only 1/216 (case 20) had positive serology for EMA and anti-TG2. He was a child of two years of age with long-term diarrhea, a background of *Rotavirus* infection, anti-TG2 levels > 14 × ULN, no HLA DQ2/DQ8, and whose EMA and anti-TG2 serology were found to be negative one month later, with no gluten restrictions. Similar cases have been reported, in which *Rotavirus* infection leads to transitory positivity for EMA and anti-TG2 [19]. 

Out of 213 non-CD patients, 198 non-CD patients were negative for both anti-TG2 and EMA, and 14 were EMA-negative (cases 6–19) but showed positive anti-TG2 levels. Among them, 5/14 children were referred from other units due to different causes (clinical symptoms suggestive of CD and/or positive anti-TG2 levels) and nine belonged to at risk groups: Three had a first-degree relative with CD, three had type 1 diabetes mellitus, and three were referred by the Rheumatology Unit because of their autoimmune diseases. Negativity of EMA, despite positivity of anti-TG2 in those 14 children (levels between 7 and 20 U/mL), could be explained because of the lower anti-TG2 specificity. Additionally, anti-TG2 presence has been observed in other clinical scenarios different from CD such as other autoimmune diseases, liver diseases, or infections. In these cases, anti-TG2 are not gluten-dependent, are in low titers, and show different specificity since anti-TG2 are not directed against CD-related TG2 epitopes but against intracellular TG2 epitopes induced by tissue damage or inflammation [20,21,22]. 

Regarding the potential CD cases, three had an autoimmune disease; in these conditions, autoantibodies develop against epitopes of the organism itself and some of them could target TG2, giving thus a rise to false positives in relation to the diagnosis of CD [23]. Three additional children with no digestive symptoms, low positive serology (EMA and anti-TG2), and no histological lesion, were siblings of confirmed CD patients. Even though they had always followed a gluten containing diet, probably gluten consumption was lower than in the general population, as shown by our group in families with CD members [24]. Although the possibility of CD development later in life cannot be ruled out, it is a matter of debate if any dietary intervention should be recommended, especially in symptomatic cases; we decided for a close follow-up approach in our patients. 

We have obtained a DOR of 12,084 in agreement with other studies (range: 64–19.397) [5]. Although it is a retrospective study, its strength lies in a large number of well-controlled patients, long lasting follow-up, as well as the clinical diagnosis being carried out by pediatric gastroenterologists, experts in CD; additionally, EMA tests were performed in a specialized laboratory by truly expert technicians. 

According to our results, and in agreement with other studies, high PPV and LR+ of EMA (99% and 209.3 respectively) make CD diagnosis more probable in a patient with positive results for these antibodies. On the one hand, different researchers give more importance to anti-TG2, even considering them as an alternative to EMA, because it is an objective, quantitative, faster, cheaper [12,25], and almost worldwide available technique [26]. On the other hand, other authors consider any of the two antibodies to be useful for CD screening as they have similar sensitivities and specificities. Although the requisite of a positive EMA when CD is diagnosed without biopsy is still a matter of debate, the ProCeDE study [2] shows that the non-biopsy approach allows for an accurate diagnosis in children and supports the use of EMA as a confirmatory test [2]. According to our results, compared to anti-TG2, additional EMA evaluation increases the diagnostic sensitivity for CD diagnosis in asymptomatic children. Likewise, IDI and NRI values obtained in our study show that a combined use of both markers displays a higher statistically significant predictive capacity, thus supporting the use of EMA for CD diagnosis, according to the ESPGHAN 2012 and 2019 guidelines [1,4]. Moreover, our results are in line with the 2019 guidelines as HLA testing is of no additional value for a serology-based diagnosis without biopsy.

## 5. Conclusions

Overall, given the wide spectrum of clinical presentation in CD, the combined use of both serological tests, EMA and anti-TG2, can contribute to a higher rate of correct classification of patients with suspected CD when a non-biopsy approach in children and adolescents is decided. 

## Figures and Tables

**Table 1:** Sensitivity and specificity, positive and negative predictive values, likelihood ratios and diagnostic odds ratio (95% confidence intervals) for EMA and anti-TG2.

	EMA+	EMA−	Anti-TG2+	Anti-TG2−
**CD patients (*n* = 232)**	228	4	229	3
**Non-CD patients (*n* = 213)**	1	212	15	198
**Sensitivity ^a^ (%)**	98.3 (95.6–99.5)		98.7 (96.3–99.7)	
**Specificity ^a^ (%)**	99.5 (97.4–100)		93.0 (88.7–96)	
**PPV ^a^ (%)**	99.6 (97.6–100)		93.9 (90.1–96.5)	
**NPV ^a^ (%)**	98.1 (95.3–99.4)		98.5 (95.7–99.7)	
**LR+ ^a^**	209.3 (29.6–1479.3)		14.0 (8.6–22.8)	
**LR− ^a^**	0.02 (0.01–0.05)		0.01 (0.00–0.04)	
**DOR ^a^**	12,084 (1339.9–10,8978.8)		1007.6 (287.5–3531.3)	

CD: Celiac disease; EMA: Anti-endomysium antibodies; Anti-TG2: Anti-tissue transglutaminase antibodies; PPV: Positive predictive value; NPV: Negative predictive value; LR+: Positive likelihood ratio; LR−: Negative likelihood ratio; DOR: Diagnostic odds ratio. ^a^ 95% Confidence Intervals are shown in parentheses.

**Table 2:** Diagnostic accuracies (95% confidence intervals) for CD diagnosis in our population considering different scenarios.

Conditions	N	TP	FP	FN	TN	Prevalence [95%CI]	Sensitivity [95%CI]	Specificity [95%CI]	PPV [95%CI]	LR+[95%CI]
**Anti-TG2 ≥10 × ULN** **EMA+** **Symptoms** **HLA compatible**	284	132	0	71	81	71.4 (65.8, 76.7)	65.0 (58.0, 71.6)	100 (95.5, 100)	100 (97.2, 100)	∞
**Anti-TG2 ≥10 × ULN** **EMA+** **Symptoms**	445	154	1	78	212	52.1 (47.4, 56.9)	66.4 (59.9, 72.4)	99.5 (97.4, 100)	99.5 (97.1, 100)	141.4 (20.0, 1001.3)
**Anti-TG2 ≥10 × ULN ** **EMA+**	445	191	1	41	212	52.1 (47.4, 56.9)	82.3 (76.8, 87.0)	99.5 (97.4, 100)	99.5 (97.1, 100)	175.4 (24.8, 1240.3)
**EMA+ ** **Symptoms**	445	181	1	51	212	52.1 (47.4, 56.9)	78.0 (72.1, 83.2)	99.5 (97.4, 100)	99.5 (97.0, 100)	166.2 (23.5, 1175.7)
**Anti-TG2 ≥10 × ULN** **Symptoms**	445	154	1	78	212	52.1 (47.4, 56.9)	66.4 (59.9, 72.4)	99.5 (97.4, 100)	99.4 (96.5, 100)	141.4 (20.0, 1001.3)
**EMA and anti-TG2 in symptomatic *versus* asymptomatic patients**										
**EMA in Asymptomatics**	109	47	0	1	61	44.0 (34.5, 53.9)	97.9 (88.9, 100)	100 (94.1, 100)	100 (92.5, 100)	∞
**EMA in Symptomatics**	336	181	1	3	151	54.8 (49.3, 60.2)	98.4 (95.3, 99.7)	99.3 (96.4, 100)	99.5 (97.0, 100)	149.5 (21.2, 1054.7)
**Anti-TG2 ≥10 × ULN** **in Asymptomatics**	109	37	0	11	61	44.0 (34.5, 53.9)	77.1 (62.7, 88.0)	100 (94.1, 100)	100 (90.5, 100)	∞
**Anti-TG2 ≥10 × ULN** **in Symptomatics**	336	154	1	30	151	54.8 (49.3, 60.2)	83.7 (77.5, 88.7)	99.3 (96.4, 100)	99.4 (96.5, 100)	127.2 (18.0, 898.2)

N: Total number of patients with data for all parameters or conditions considered in the specific scenario; TP: True positive; FP: False positive; FN: False negative; TN: True negative; EMA: Anti-endomysium antibodies; Anti-TG2: Anti-tissue transglutaminase antibodies; PPV: Positive predictive value; LR+: Positive likelihood ratio. 95% Confidence Intervals are shown in parentheses.

## Supplementary Materials

The following are available online at https://www.mdpi.com/2077-0383/8/12/2179/s1, Table S1: Descriptive analysis of the population.

## Abbreviations

Anti-DGP	Deamidated gliadin peptide antibodies
AGA	Anti-gliadin antibodies
Anti-TG2	Anti-tissue transglutaminase antibodies
CD	Celiac Disease
CI	Confidence interval
DM	Diabetes mellitus
DOR	Diagnosis odds ratio
EMA	Anti-endomysium antibodies
ESPGHAN	European Society for Paediatric Gastroenterology, Hepatology and Nutrition
FITC	Fluorescein isothiocyanate
FN	False negative
FP	False positive
GFD	Gluten free diet
HLA	Human leukocyte antigen
IDI	Integrated Discrimination Improvement
IFI	Indirect immunofluorescence
IgA	immunoglobulin A
LR-	Negative Likelihood ratio
LR+	Positive Likelihood ratio
ND	No data
NPV	Negative predictive value
NRI	Net Reclassification Improvement
PCR	Polymerase chain reaction
PPV	Positive predictive value
SIB	Small intestinal biopsy
SD	Standard deviation
TG2	Tissue transglutaminase type 2
TN	True negative
TP	True positive
ULN	Upper limit of normal
