# Supplementary material for: Efficacy Study of Anti-Endomysium Antibodies for Celiac Disease Diagnosis: A Retrospective Study in a Spanish Pediatric Population
Source: J Clin Med. 2019 Dec 11;8(12):2179. doi: 10.3390/jcm8122179 (PMC6947542; doi:10.3390/jcm8122179)
Supplement: Supplementary file 1 [file jcm-08-02179-s001.pdf]

**Supplemental Table 1**. Descriptive analysis of the population.

| | **Group 1 (n=232)** | | | **Group 2 (n= 213)** | | |
|---|---|---|---|---|---|---|
| **Diagnosis** | CD | | | Non CD | | |
| **Mean age (SD) in years** | 5.6 (3.9) | | | 6.7 (4.1) | | |
| **EMA** | 228 + | | 4 - * | 1 + | 212 - | |
| **Anti-TG2** | 227 + | 1 - | 2+ / 2- ** | 1 + | 14 + | 198 - |
| **HLA DQ2/DQ8** | 197 (28ND) | 1 | 4 | - | 10 (4ND) | 63 (124ND) |
| **With symptoms** | 178 | 1 | 3 | 1 | 7 | 145 |

CD: celiac disease; EMA: anti-endomysium antibodies; Anti-TG2: anti-tissue transglutaminase antibodies; HLA: human leukocyte antigen; ND: no data; SD: standard deviation.

* 4-= 4 had a negative result for EMA; ** 2+/2- out of these 4-, 2 were anti-TG2 positive and 2 negative.
